# Supplementary material for: Expectancy-related changes in firing of dopamine neurons depend on hippocampus
Source: Nat Commun. 2024 Oct 16;15:8911. doi: 10.1038/s41467-024-53308-z (PMC11484966; doi:10.1038/s41467-024-53308-z)
Supplement: Supplementary file 1 — Supplementary Information [file 41467_2024_53308_MOESM1_ESM.pdf]

Supplementary Information

Supplementary Methods:

Modeling with free-choice odor:

To simulate the prediction error evoked by the free-choice odor, we introduced four additional states corresponding to the presentation of the free-choice odor in four blocks and one additional observation to represent the onset of the free-choice odor. The transition probabilities to these states were set the same as those for the forced-choice odor states, and the dwell distribution of these states was identical as well. Unlike the forced-choice odor, the free-choice odor states are allowed to transit to both left and right well states within the same block. After transitioning to the left well states, the transition probabilities to the left well states are 1. Similarly, after transitioning to the right well states, the transition probabilities to the right well states are 1. This ensures that the model can accurately identify the current well state.

The expected value of the free-odor cue states equals the sum of their action values weighted by the probability of choosing each action (Eq. 1).

$$\boldsymbol{value_{free} = value_{left} * p(left) + value_{right} * p(right)}, \quad (1)$$

$\boldsymbol{value_{left}}$ and $\boldsymbol{value_{right}}$ are the expected values of choosing the left well and right well, respectively. $\boldsymbol{p_{\mathrm{left}}}$ and $\boldsymbol{p_{\mathrm{right}}}$ are the probabilities of choosing the left well and right well, respectively, and are determined by the estimated block. $\boldsymbol{p_{\mathrm{left}}}$ is calculated as the sum of probabilities that the model is in the blocks where left well is the high-value option. $\boldsymbol{p_{\mathrm{right}}}$ equals $\boldsymbol{1 - p_{\mathrm{left}}}$.

Supplementary Figures:

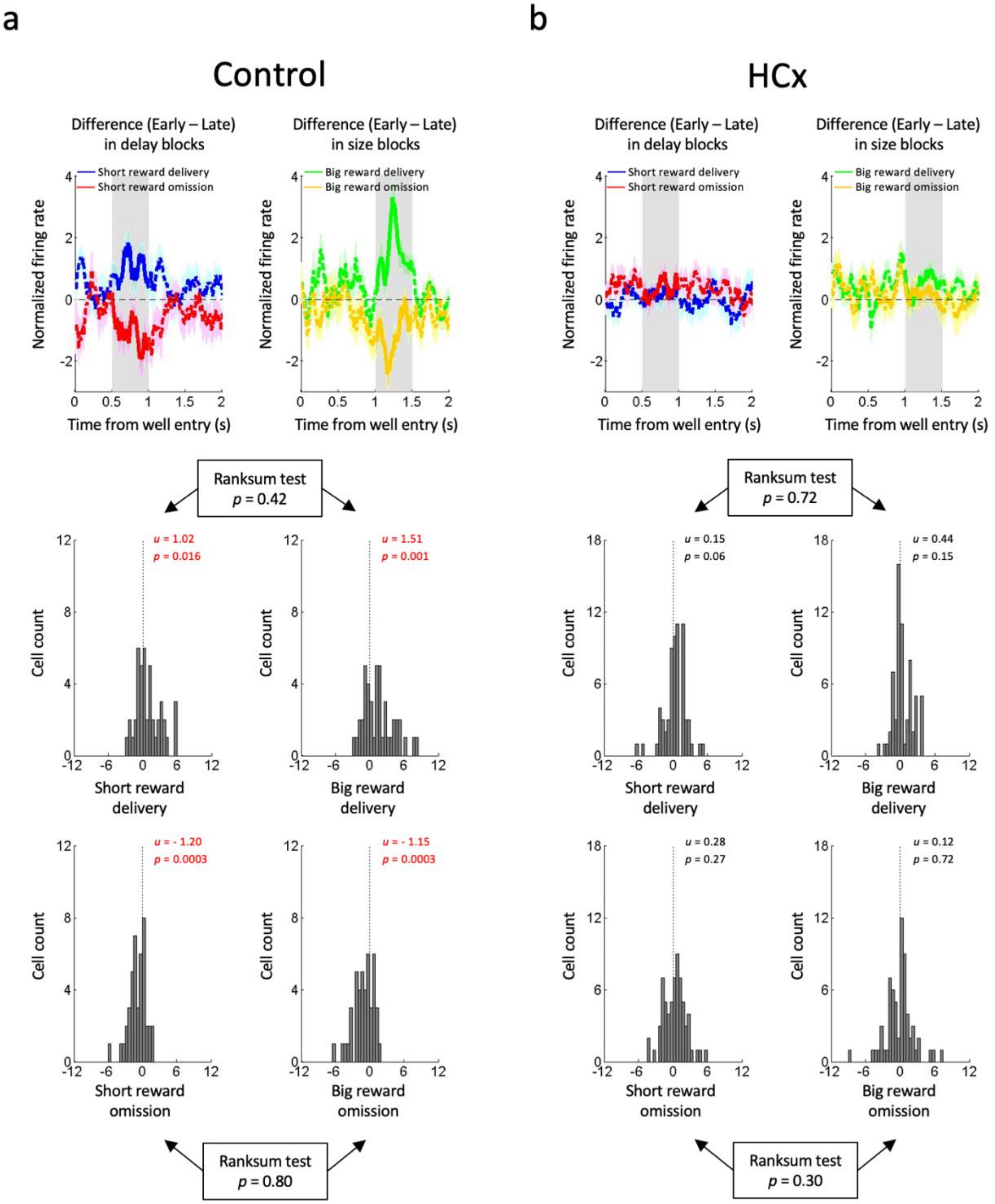

Supplementary Figure 1. The dopamine response caused by both the delay and size switches in control and HCx rats. **(a)** In the control rats, both the change in reward delay (left panels) and size (right panels) induce the dopamine response (n=44), and this response dismisses over learning. The numbers of neurons significantly

affected by the delay and size changes are not significantly different. **(b)** In the HCx rats, neither the change in reward delay (left panels) nor in the reward size (right panels) induce the dopamine response (n=66). Data in panels (a-b) are presented as mean values +/− S.E. The numbers of neurons significantly affected by the delay and size changes are not significantly different. The numbers in each panel indicate results of two-sided Wilcoxon singed-rank test (p) and the average difference score (u). Significant effects are indicated in red.

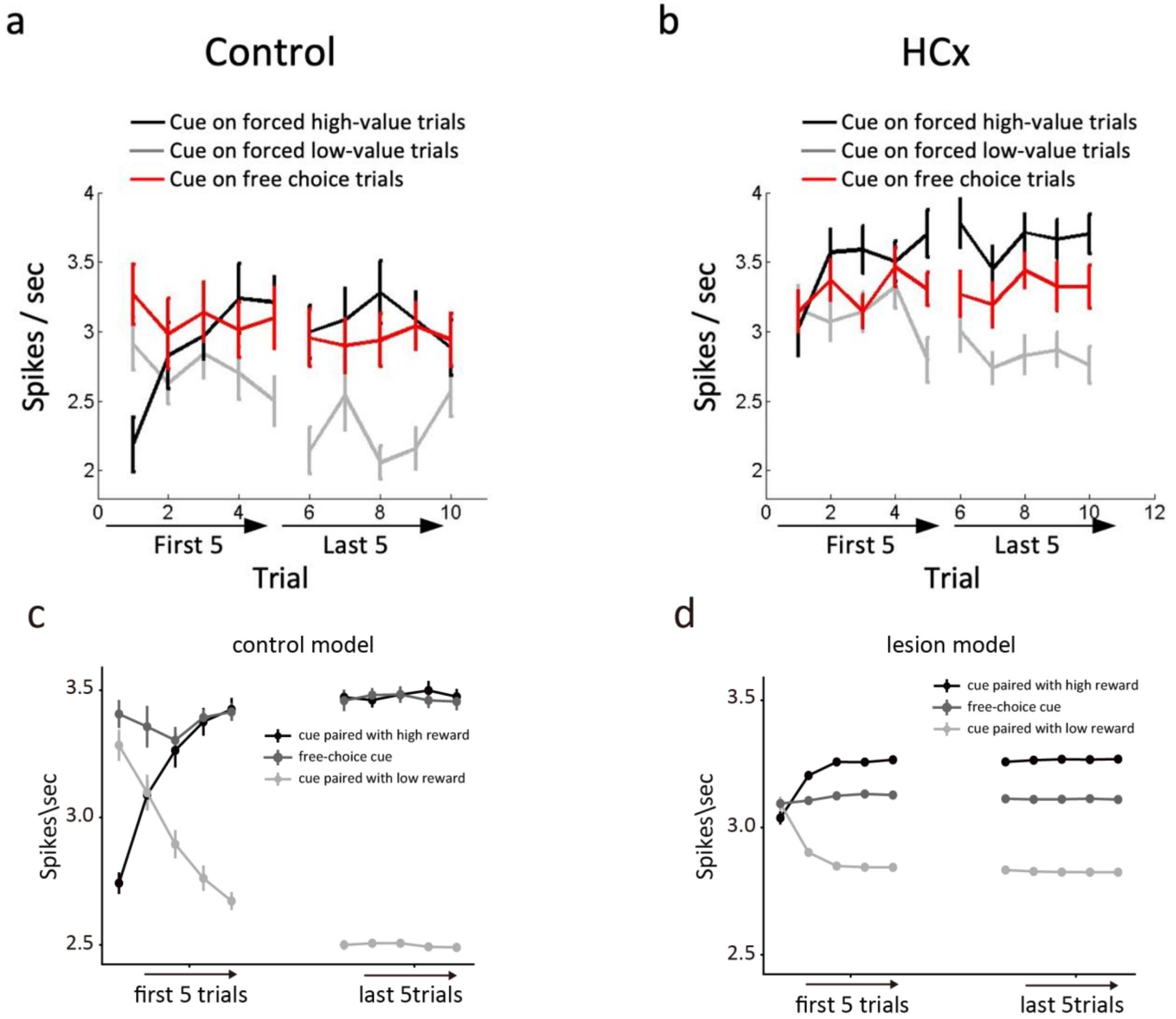

Supplementary Figure 2. Cue-evoked dopamine response in free-choice trials control and HCx rats. **(a)** In the control rats, the firing of DA neurons in response to the free-choice cue (red line) reflected the more variable option (black line; ANOVA, p=0.51). **(b)** In the HCx rats, the firing response to the free-choice cue (red line) in later trials was significantly lower than that to the high-valued cue (black line; ANOVA, p=0.002) and significantly higher than that to the low-valued cue (grey line; ANOVA, p=0.011). (**c**) Simulated average cue-evoked prediction errors in the control model with hierarchical task space. The black and grey lines represent the prediction error in response to odor cues paired with high and low reward, respectively. The dark grey line represents the prediction error in response to odor cues indicating free choice. **(d)** The format is the same as supp Fig. 2c, but for the lesion model. Data in all panels are presented as mean values +/− S.E.

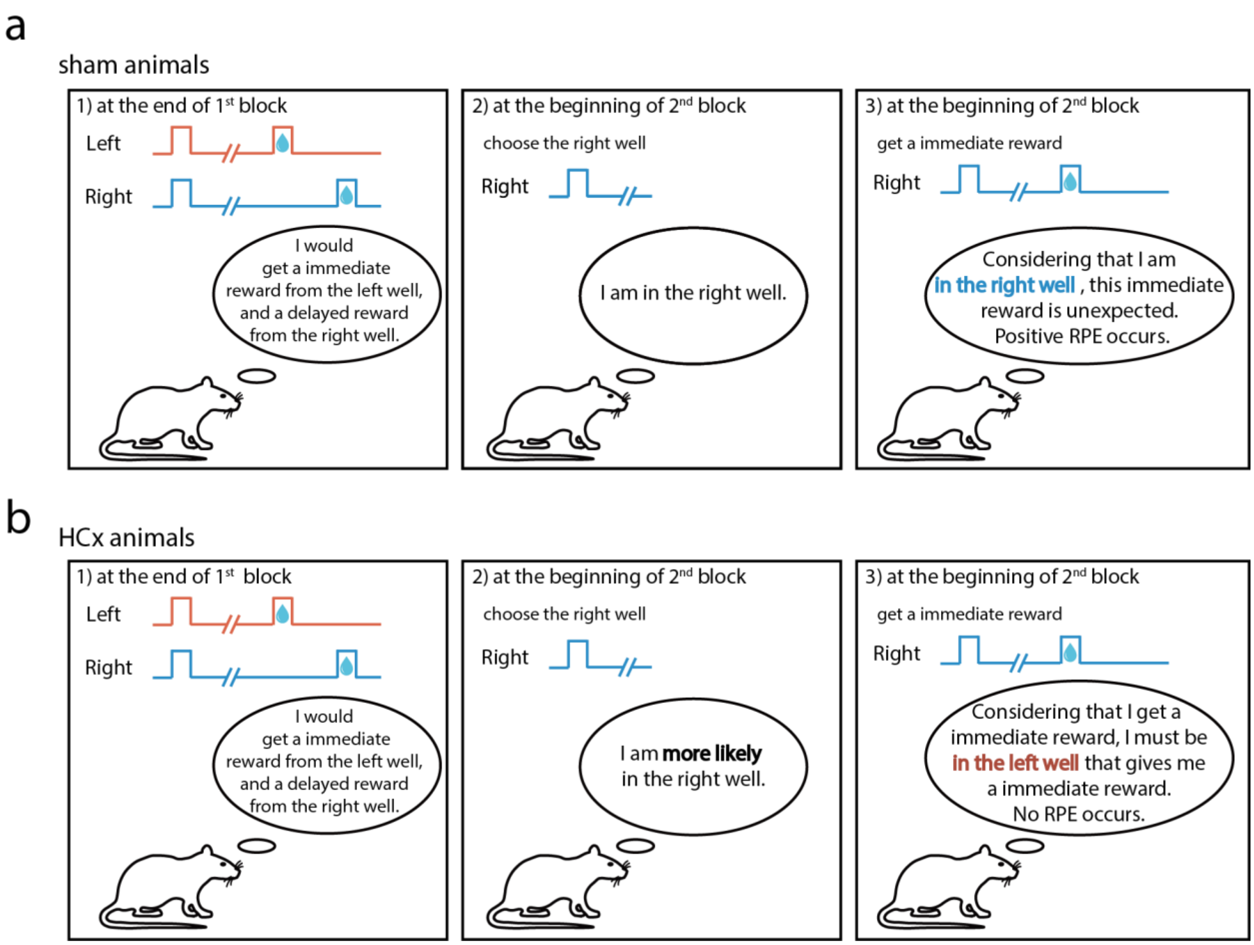

Supplementary Figure 3. An example showing what states the model with the flat state space confuses. **(a)** The intact model has a clear understanding of the current state, and the unexpected reward delivery at the beginning of the 2nd block does not change the state estimation but induces a prediction error. **(b)** The lesion model has a larger uncertainty in estimating the states between left and right well states, and the unexpected reward delivery at the beginning of the 2nd block changes the state estimation to match the observations, resulting in no prediction error.

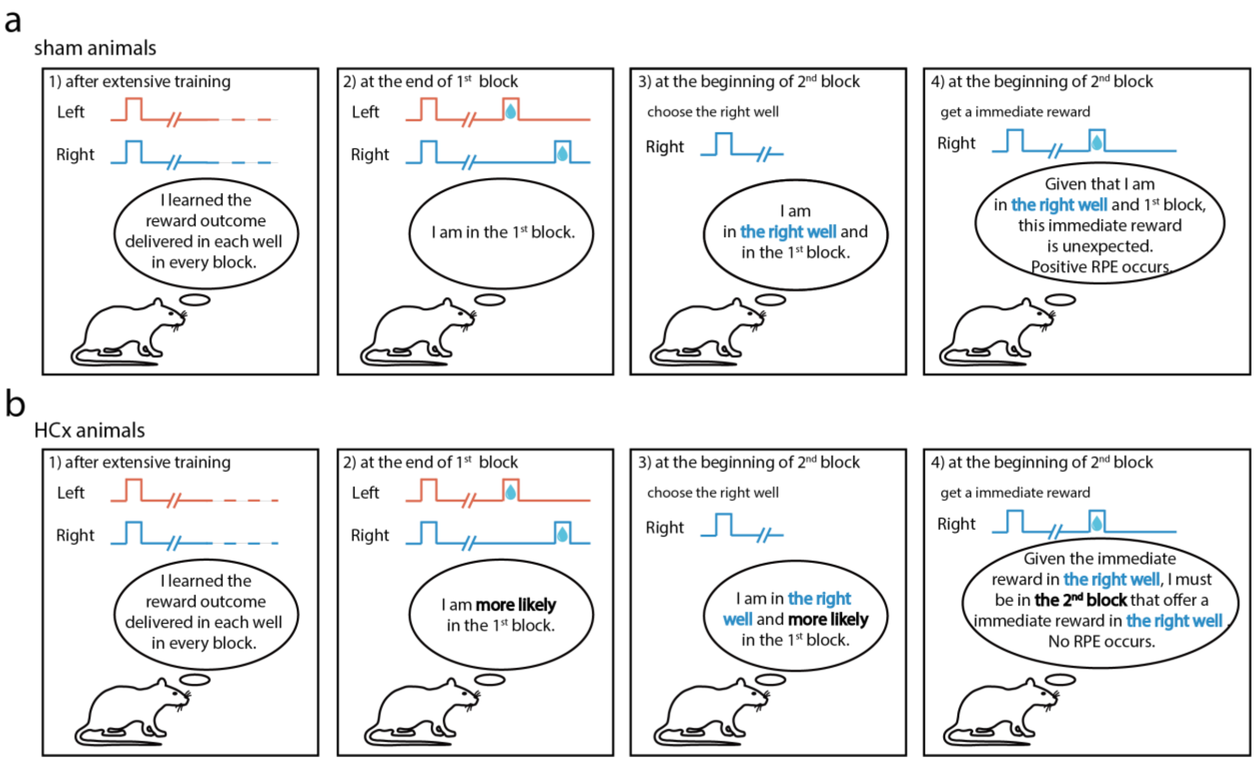

Supplementary Figure 4. An example showing what states the model with the hierarchical state space confuses. **(a)** The intact model clearly estimates the current block, and the unexpected reward delivery at the beginning of the 2nd block does not change the estimation in the current state and block but induces a prediction error. **(b)** Greater uncertainty is introduced in the lesion model when estimating the current block. Consequently, the unexpected reward delivery at the right well at the beginning of the 2nd block shifts the state estimation from the right well state in the 1st block to the right well state in the 2nd block to align with the observations, thereby resulting in no prediction error.

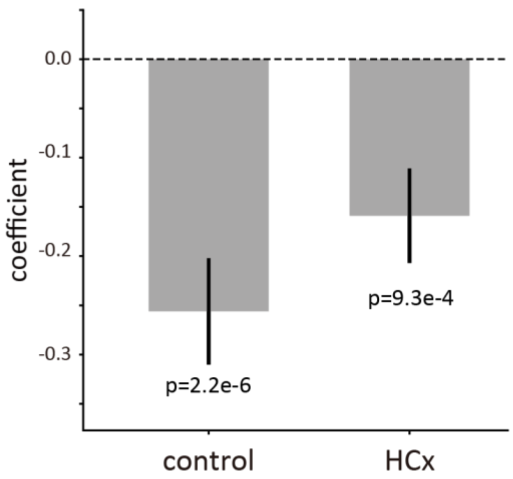

Supplementary Figure 5. After switching to the 2nd and 4th blocks, rats received unexpected rewards at both wells. In the flat model, these unexpected rewards only modify the value of entering that well, while in the hierarchical model, an unexpected reward in one well can modify the value of reward in the other well, using knowledge of the task structure. However, this knowledge is not unambiguous. Due to quirks in the block design, when an immediate or small reward is received, the model predicts that the other well contains either a big or a delayed reward, which leaves some ambiguity since these two rewards can be either more or less advantageous. By contrast, when a big or delayed reward is received, the model confidently predicts an immediate/small reward in the other well, eliminating uncertainty about the reward outcomes associated with both wells. Therefore, the hierarchical model is able to make better judgments about which well is associated with a better reward after receiving a big or delayed reward, than after receiving an immediate/small reward. In contrast, the flat model cannot make cross-well predictions based on the task structure, instead treating each well independently without. Consequently, the flat model would not be expected to show any difference in predictions after receiving a big reward versus after receiving a small reward. To determine which model's prediction is consistent with the observed behavior, a logistic regression is used to and measure the effect of the reward feedback from the previous trial on the choice in free-choice trials. The model includes a constant and one variable: $reward$=1 or 0 when rats receive an immediate/small reward or a big/delayed reward in the previous trial, respectively. The dependent variable y=1 or 0 when rats choose the high-valued or low-valued side in the current free-choice trials, respectively. In the regression, data from all rats and all sessions are combined. Two-sided t-tests were used to evaluate the significance of the coefficients. In both control (n=6092) and HCx (n=7375) groups, variable $reward$ significantly affected rats' choices in the free-choice trials. Data are presented as mean values +/− S.E.

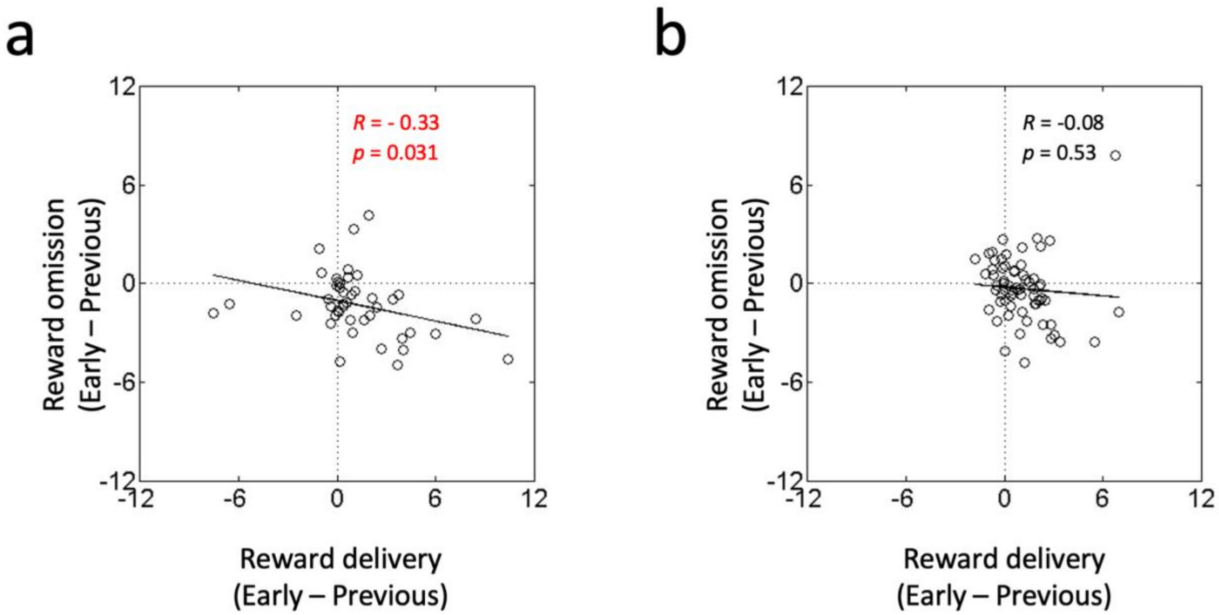

Supplementary Figure 6. The relationship between the positive and negative prediction errors at the single neuron level in the control and HCx rats. (**a**) The change in normalized firing (n=44), which is the average normalized firing in late trials subtracted from that in early trials, in response to reward delivery correlates with that in response to reward omission in control rats. (**b**) This correlation was not found in HCx rats (n=66). The numbers in each panel indicate results of two-sided Wilcoxon singed-rank test (p) and the average difference score (u). Significant effects are indicated in red.
